# Research on design and control methods of a lightweight upper limb joint isokinetic rehabilitation training equipment

**DOI:** 10.3389/fbioe.2024.1430372

**Published:** 2024-09-03

**Authors:** Zhou Zhou, Yuzhu Wan, Yingbing Su, Yunwei Li, Bingshan Hu, Hongliu Yu

**Affiliations:** ^1^ Institute of Rehabilitation Engineering and Technology, University of Shanghai for Science and Technology, Shanghai, China; ^2^ Aerospace System Engineering Shanghai, Shanghai, China; ^3^ Shanghai Engineering Research Center of Assistive Devices, Shanghai, China

**Keywords:** rehabilitation robot, mechanism design, active disturbance rejection, nonlinear control, isokinetic, joint torque

## Abstract

**Introduction:**

Isokinetic exercise can improve joint muscle strength and stability, making it suitable for early rehabilitation of stroke patients. However, traditional isokinetic equipment is bulky and costly, and cannot effectively avoid external environmental interference.

**Methods:**

This paper designed a lightweight upper limb joint isokinetic rehabilitation training equipment, with a control system that includes a speed planning strategy and speed control with disturbance rejection. Based on the established human-machine kinematic closed-loop model between the equipment and the user, a dynamic evaluation method of torque at the joint level was proposed.

**Results:**

To validate the effectiveness of the equipment, experiments were conducted by manually applying random disturbances to the equipment operated at an isokinetic speed. The results showed that the root mean square error between the observed torque curve of the second-order linear extended state observer used in this paper and the actual disturbance curve was 0.52, and the maximum speed tracking error of the speed control algorithm was 1.27%. In fast and slow sinusoidal speed curve tracking experiments, the root mean square errors of the speed tracking results for this algorithm were 9.65 and 5.27, respectively, while the tracking errors for the PID speed control algorithm under the same environment were 19.94 and 12.11.

**Discussion:**

The research results indicate that compared with traditional PID control method, the proposed control strategy demonstrates superior performance in achieving isokinetic control and suppressing external disturbances, thereby exhibiting significant potential in promoting upper limb rehabilitation among patients.

## 1 Introduction

Stroke is currently one of the most common causes of physical disabilities in the world (Feske, 2021), and it is difficult to cure, with most patients potentially experiencing hemiplegia (Dobkin, 2004). Hemiplegic individuals commonly face issues such as dyskinesia, sensory impairment, and difficulty walking, leading to a decrease in their activities of daily living (Sun et al., 2021). Research has shown that early bedside exercise rehabilitation for stroke patients has a positive impact on reducing the consequences of the disease (Lee et al., 2015). Among various forms of sports training, isokinetic exercise can achieve the maximum muscle training effect at any joint angle, and can also improve joint stability and endurance, making it suitable for early sports rehabilitation training (Huang et al., 2003).

Isokinetic exercise requires the use of isokinetic muscle strength training equipment (hereinafter referred to as isokinetic equipment). Isokinetic equipment is a professional rehabilitation equipment that can improve dyskinesia, comprehensively exercise joint muscle strength, endurance, stability, and quantitatively estimate torque at the joint level (Sin et al., 2018; Pontes et al., 2019). Isokinetic equipment has great scientific research value and application prospects in the field of rehabilitation training (Wang et al., 2023).

Among the current isokinetic equipment, well-known ones include Cybex (Lin et al., 2008), Biodex (Lund et al., 2005), ISOMED (Roth et al., 2017), etc. However, these commercial products are bulky and costly. Other isokinetic equipment solutions have been proposed by researchers. In early research, isokinetic equipment was mostly implemented using hydraulic, magnetorheological braking, and other actuating methods, for example, Oda et al. proposed an isokinetic contraction motion equipment based on magnetorheological fluid brakes (Oda et al., 2009); Kikuchi developed a braking device using granular electrorheological fluid and applied it to isokinetic rehabilitation training (Kikuchi et al., 2003). However, it has been found that there are drawbacks such as complex structure and heavy weight in the design using the aforementioned actuating method.

With the development of electric motors and its control technologies, more and more researchers have proposed the design scheme of isokinetic equipment using motors for direct actuating, for example, Bae developed an isokinetic motion experimental equipment for elbow joints using an elastic actuator actuated by a motor (Bae et al., 2019); Dhandapani designed an isokinetic shoulder equipment for postoperative rehabilitation using a DC motor (Dhandapani et al., 2023); Sheng and Guo used a fuzzy PID controller to control the isokinetic motion of a brushless DC motor, achieving excellent stability and adaptability (Sheng and Liu, 2019; Guo et al., 2022). The isokinetic equipment designed based on the motor not only achieves the goal of isokinetic rehabilitation, but also has the advantage of being lighter in weight and having a simpler structure, making it suitable for the development of isokinetic equipment.

One of the key technologies of isokinetic equipment is the research on high-precision speed control algorithms. Scholars have proposed speed control algorithms such as fuzzy control (Gowthaman et al., 2017; He et al., 2020) and adaptive control (Feng and Jiao, 2017; Jon et al., 2017). However, in rehabilitation training, the external force exerted by the user on the equipment is uncertain and consequently the above algorithm is not suitable for speed control of isokinetic equipment. The active disturbance rejection control (ADRC) has the advantages of high control accuracy, strong anti-interference ability, and low dependence on the model (Han, 2009; Chen and Yang, 2023). The nonlinear active disturbance rejection control (NLADRC) algorithm, which integrates extended state observers, can handle disturbances in the system that cannot be accurately modeled, thus this method is suitable for speed control of isokinetic equipment (Gao, 2014).

Another key technology of isokinetic equipment is the quantitative estimate of torque at the joint level. During continuous dynamic testing of the joint, isokinetic joint torque estimate can quantify the torque at the joint level without subjective influence from the observer, and the quantitative data of its relevant evaluation indicators plays a significant role in the formulation of rehabilitation plans (Yoon et al., 2021; Daumas et al., 2023). At present, the commonly used estimate indicators for isokinetic technology include peak torque, average joint range of motion, average power, etc. Among them, peak torque is called the golden indicator (Huang et al., 2013). Scholars have adopted two main approaches for the recognition of human-machine interaction torque: sensor-based and biometric signal detection-based (Wang et al., 2022; Kizyte et al., 2023). While the biometric signal-based method is more accurate, it requires additional biometric signal detection equipment, which adds inconvenience of wearing and production cost (Zhou et al., 2019). In contrast, methods based on direct measurement by torque sensors have some limitations, such as including gravitational torque, inertial torque, etc., and cannot be directly used as the results of isokinetic joint torque estimate. To address the aforementioned issues, this paper takes the elbow joint as an example to establish a human-machine mechanical closed chain model between isokinetic equipment and trainers, and proposes a calculation method for dynamic joint torque, which serves as the foundation for estimating torque related indicators.

The contributions of this work are presented as follows: Firstly, considering the need for mobility, a low-cost lightweight upper limb joint isokinetic rehabilitation training equipment is designed. Secondly, starting from the requirement of isokinetic training, the speed planning is conducted using motion intention analysis based on joint torque, and a second-order nonlinear active disturbance rejection speed control method is adopted to achieve isokinetic motion in accordance with human movement, and to improve the disturbance rejection capability and robustness of the speed controller. Furthermore, by establishing a human-machine kinematic closed-loop model between the isokinetic equipment and the user, a dynamic evaluation method of torque at the joint level is proposed, effectively eliminating system errors in torque estimation.

The rest of this paper is organized as follows. The second section introduces the lightweight upper limb joint isokinetic rehabilitation training equipment designed. It covers two aspects, structure design and control system software architecture design. The third section introduces the speed planning method based on motion intention recognition and the speed control method based on second-order NLADRC. The fourth section introduces the proposed dynamic estimate method of torque at the joint level. In the fifth section, the equipment was tested for overall operation, and the effects of its speed control and joint torque estimation effects were verified and analyzed; The conclusion is drawn in the final section.

## 2 Design of lightweight upper limb isokinetic rehabilitation equipment

The lightweight isokinetic equipment designed by this research is primarily intended for upper limb rehabilitation training. Considering the range of motion of the upper limb joints and the demand for isokinetic equipment, the lightweight isokinetic equipment is designed as a single degree of freedom structure. This section provides a detailed introduction to the designed lightweight isokinetic equipment from two aspects, structural design and control system software architecture design.

### 2.1 Structural design

**FIGURE 1:**
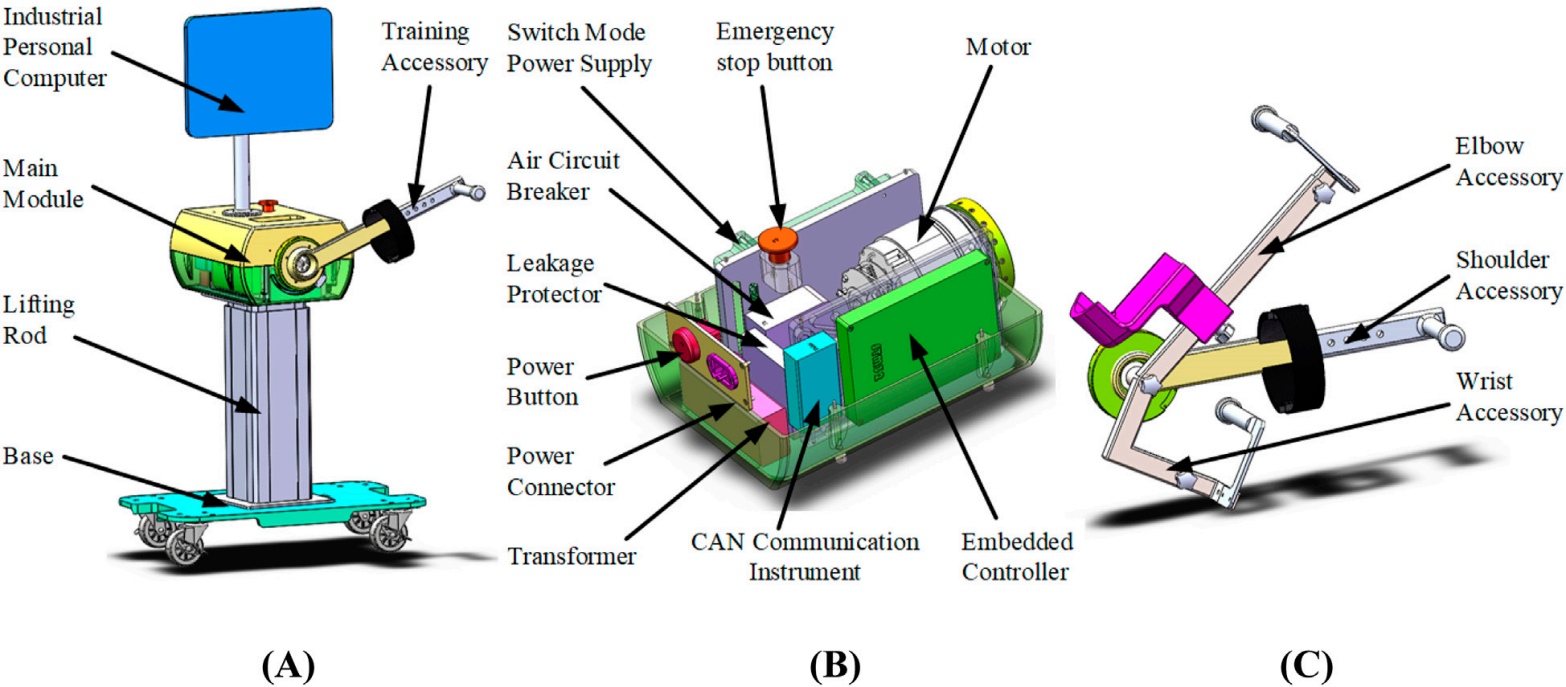
Isokinetic equipment model diagram. **(A)** Appearance model diagram **(B)** Internal structure of the main module **(C)** Training accessory model diagram.

The appearance model of the lightweight isokinetic equipment is shown in Figure 1A. The main structure of the equipment includes a base, lifting rod, main module, industrial personal computer (IPC), and training accessory. Considering the application scenario of lightweight isokinetic equipment that requires easy mobility to meet special rehabilitation training needs such as bedside rehabilitation, four universal wheels have been integrated to design the base of this equipment. The length of the lifting rod can be adjusted to adapt to different working environments. The IPC serves as the control interface for human-machine interaction and the visualization port for isokinetic training data.

The internal structure of the main module of the isokinetic equipment is shown in Figure 1B, consisting of switching mode power supply, emergency stop button, motor, air circuit breaker, leakage protector, power button, power connector, transformer, CAN communication instrument, and embedded controller (EC). The rated working voltage of the equipment is 220 V from the mains supply. When the mains supply is connected, one circuit of electricity is reduced through a transformer to supply power to the IPC, and the other circuit of electricity is rectified and filtered by a switching mode power supply and converted into direct current to supply power to other devices. To ensure the safety of equipment operation, air circuit breaker and leakage protector are integrated to ensure the electrical safety of the equipment, and an emergency stop button is integrated to prevent dangerous work from occurring. The motor adopts Tech Robots RJSIZ-20 joint module, with a rated speed of 29.7 rpm and a rated torque of 61 Nm. Its maximum instantaneous torque can reach 182 Nm. To achieve closed-loop control of equipment motion output, the motor adopts a dual encoder structure, where an incremental encoder with a resolution of 20,000 P/R is used for motor operation control, and a 17 bit absolute value encoder is used for joint output attitude control.

The human body is coupled with isokinetic equipment through training accessories. In order to meet the training needs of different patients and joints, training accessories are divided into three categories, namely, elbow accessory, shoulder accessory, and wrist accessory, as shown in Figure 1C. They are connected to isokinetic equipment through an easily detachable interface, corresponding to the rehabilitation training of users' shoulder pitch, elbow pitch, and wrist pitch degrees of freedom. There are different patients who have different body height dimensions, and in order to better adapt to their training needs, the relative positions of various components of the training accessories can be adjusted.

### 2.2 Control system software architecture design

**FIGURE 2:**
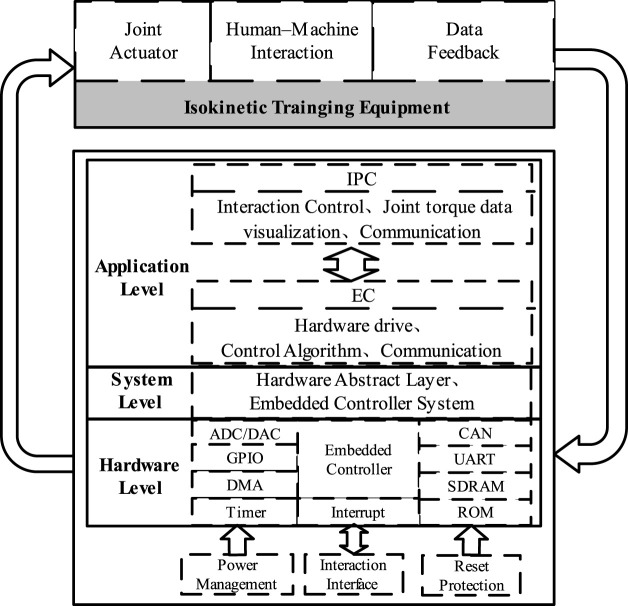
Isokinetic equipment software architecture.

Based on the system requirements of isokinetic equipment and considering that simpler coupling relationship between modules in software design, the easier the implementation and maintenance of the system, the isokinetic equipment software system is divided into underlying hardware driver control module, isokinetic equipment control algorithm implementation module, communication module, as well as interaction control module, joint torque estimation module, and data visualization module. According to the implementation method of the module, it is arranged in the EC and IPC, and the software architecture of the isokinetic equipment is shown in Figure 2.

IPC utilizes joint torque data visualization software to achieve interactive control of isokinetic equipment and joint torque data visualization. The interactive control module is used to achieve the human-machine interaction function of the equipment, using buttons, and text input and output to achieve the operation control of isokinetic equipment and communication settings of EC. The visualization of joint torque data enables the presentation of real-time speed and estimate result of torque at the joint level in the form of real-time curves. The communication module of IPC achieves data transmission with the EC through UART.

EC is used to implement the underlying hardware drivers and control algorithms. The underlying hardware driver control module realizes the drive of basic peripherals, while integrating the CANopen protocol stack to achieve torque control of the motor and sensor data collection. After decoupling between each module, a unified communication interface is used, and the communication module implements CAN communication and UART communication according to standard protocols.

## 3 Speed control algorithm

**FIGURE 3:**
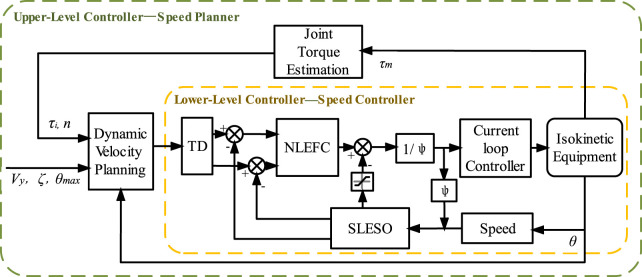
Isokinetic equipment control system block diagram.

A speed planning strategy based on estimation of torque at the joint level was adopted to achieve isokinetic training of the equipment under human motion compliance, solving the impact problem of equipment during the acceleration and deceleration stages in rehabilitation exercises. Secondly, considering the operating environment of isokinetic equipment, second-order NLADRC was adopted to avoid the influence of disturbances and improve the anti-interference ability and robustness of the speed controller. The block diagram of the isokinetic equipment control system is shown in Figure 3, which consists of an upper-level speed planner and a lower-level speed controller.

The speed planner consists of a joint torque estimation module, a dynamic speed planning module, and a speed controller. The joint torque estimation module adopts the method proposed in Section 4 to dynamically estimate the torque at the joint level $\tau_{i}$ based on the external torque $\tau_{m}$ applied to the equipment. Based on expected training speed $V_{y}$ and expected damping coefficient ζ , equipment working angle θ , preset maximum working angle $\theta_{\max}$ , and joint torque estimation result $\tau_{i}$ , the dynamic speed planning achieves the recognition of the user’s movement intention and the compliant movement of the equipment. In the acceleration and deceleration stages, the acceleration of the equipment increases with higher joint torque $\tau_{i}$ . However, once the actual operational speed of the equipment reaches the expected training speed $V_{y}$ , regardless of the magnitude of the joint torque $\tau_{i}$ , the equipment will maintain the expected speed $V_{y}$ until there is an intent from the user to initiate a reverse movement.

The speed controller adopts the NLADRC algorithm framework and achieves equipment output control through current loop. For the speed control of isokinetic equipment, a tracking differentiator (TD) is used to reduce the overshoot, and a second-order linear extended state observer (SLESO) is used to reduce the nonlinear time-varying interference generated during human-machine interaction and the influence of internal uncertainty in the equipment. For cases where sudden changes in signals result in excessive disturbance estimation results, saturation limiting filtering is applied, and nonlinear error feedback control (NLEFC) is used for adaptive control to achieve high-quality control of isokinetic equipment. Finally, stability analysis was conducted on the SLESO and speed controller.

### 3.1 Speed planner

**FIGURE 4:**
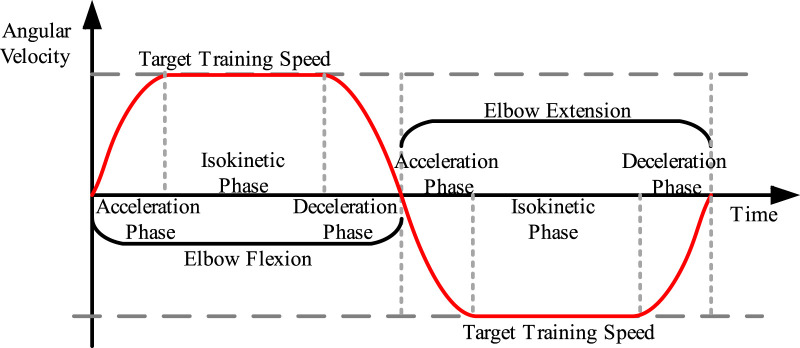
Speed planning for isokinetic equipment.

In isokinetic rehabilitation training, the range of human activity is limited. In order to achieve sustained movement, the equipment’s movement is designed as a single joint reciprocating rotational motion. According to the ideal isokinetic motion curve, the speed of the equipment can be planned into acceleration, deceleration, and isokinetic motion stages. The speed planning of the isokinetic equipment is shown in Figure 4.

Define the expected flexion and extension intention of the user’s joint as. The Q value is 1 when the user wants to flex their joint, −1 when they want to extend their joint, and 0 when they don't want to move. Based on the $M_{j}$ obtained in Section 4, which is the torque at the joint level, the following formula (Equation 1) can be derived:

$$Q=\left\{\begin{array}{cc} 1, & M_{j}\geq M_{thr}\\ 0, & -M_{thr}<M_{j}<M_{thr}\\ -1, & M_{j}\leq -M_{thr} \end{array}\right.$$ (1)

In the formula, $M_{thr}$ represents the torque threshold, which is a pre-defined parameter with a value of 0.6 Nm. Taking into account measurement errors and environmental factors affecting equipment operation, minor fluctuations in the identified torque may occur. Effective torque is recognized only when the user’s joint torque exceeds this threshold.

The magnitude of equipment acceleration is related to the magnitude of torque at the joint level. During the acceleration or deceleration stages, the user’s intention is directly mapped to the speed input of the equipment, allowing the equipment to move according to the user’s intention. Defining $\tau_{i}=\left|M_{j}\right|$ , At this point, the expected training speed is (Equation 2):

$$V_{u}=\begin{array}{cc} C\tau_{i}Q, & C\tau_{i}<V_{y} \end{array}$$ (2)

In the equation, $V_{u}$ is the input value for equipment speed control, at which point $V_{u}$ is less than the target training speed $V_{y}$ . C , the velocity mapping coefficient, reflects the rate at which the equipment adapts to the user’s joint torque. As the C value increases, the speed compliance of the equipment becomes stronger and the damping effect is smaller.

When the speed $V_{u}$ reaches the target training speed $V_{y}$ , no matter how big interaction torque $\tau_{i}$ is, the planned speed $V_{u}$ will remain unchanged. The focus at this stage is on the user’s muscle endurance and strength training. There is the following formula (Equation 3):

$$V_{u}=V_{y}Q$$ (3)

The final input function for equipment speed control (Equation 4) can be obtained by combining Equation 2 and Equation 3:

$$V_{u}=\left\{\begin{array}{cc} C\tau_{i}Q, & C\tau_{i}<V_{y}\\ V_{y}Q, & C\tau_{i}\geq V_{y} \end{array}\right.$$ (4)

In practical applications, it is necessary to restrict the angular displacement of the isokinetic equipment, as the user’s joint have a rotation angle range, and exceeding this range may indicate an abnormal situation. The following angle restrictions apply (Equation 5):

$$\left|\theta -\theta_{0}\right|<\theta_{\max}$$ (5)

In the equation, $\theta_{\max}$ is the maximum rotation angle, $\theta_{0}$ is the initial position.

### 3.2 Speed controller

This paper examines the utilization of NLADRC in the speed control of isokinetic equipment. Unlike traditional control methods that rely on precise system models, NLADRC operates based on real-time speed commands and feedback data of equipment speed. During operation, the transition process is configured using the TD. NLEFC is employed to adjust the control gain, and a SLESO is utilized to estimate the disturbance in real time. Then, the observed disturbance is compensated through feedforward control. Finally, output torque control commands are generated to govern the motor’s operation.

#### 3.2.1 Tracking differentiator

The isokinetic control achieved in the traditional speed regulation process may encounter problems such as insufficient speed or excessive overshoot under the influence of factors such as structure inertia and time delay. However, by combining the TD with the optimal fast comprehensive control function, it is possible to achieve the optimal configuration of the transition process (Han, 2009). This not only helps in smoothing the output differential signal but also ensures minimal overshoot and fast tracking.

The discrete form of TD is (Equation 6):

$$\left\{\begin{array}{c} \;F_{fs}=F_{fast}\left(s_{1}\left(k\right)-V_{u}\left(k\right),s_{2}\left(k\right),r,h_{0}\right)\\ s_{1}\left(k+h\right)=s_{1}\left(k\right)+hs_{2}\left(k\right)\;\\ s_{2}\left(k+h\right)=s_{2}\left(k\right)+hF_{fs}\; \end{array}\right.$$ (6)

In the formula, $F_{fast}\left(\right)$ is the optimal fast comprehensive control function, $s_{1}$ is the introduced transition velocity, $s_{2}$ is the differential form of $s_{1}$ , r is the speed factor, $h_{0}$ is the filtering factor, and h is the step size. According to experience, it is more suitable when $h_{0}=5h$ .

#### 3.2.2 Second-order linear extended state observer

During the human-machine interaction process of isokinetic equipment, the unstable factors inherent in the equipment and external disturbances caused by humans can seriously affect the operation of the equipment. In the process of speed control, it is necessary to use SLESO to calculate the disturbance from the input and output information of isokinetic equipment, and then perform feedforward compensation to improve the control effect.

The control system for isokinetic equipment can be simplified as a second-order system as follows (Equation 7):

$$\{\begin{array}{l} \ddot{x_{1}}-a_{1}\dot{x_{1}}-a_{2}x_{1}=w+\psi u\left(t\right)\\ y=x_{1}\; \end{array}$$ (7)

In the equation, $a_{1}$ and $a_{2}$ are parameters of the isokinetic system, w is the external disturbance, ψ is the compensation coefficient of the controller, u is the output of the speed controller, y is the feedback speed input.

Define the total disturbance received by the system as $F_{d}=\ddot{x_{1}}$ , and extend it to the system state variable $x_{3}$ . Obtain the state equation of the system (Equation 8):

$$\left\{\begin{array}{c} \dot{x_{1}}=x_{2}\;\\ \dot{x_{2}}=x_{3}+\psi u\\ \dot{x_{3}}=\dot{F_{d}}\; \end{array}\right.$$ (8)

Establish a second-order linear extended state observer (Equation 9):

$$\left\{\begin{array}{c} \dot{z_{1}}=z_{2}-\;\beta_{1}\varepsilon_{1}\;\\ \dot{z_{2}}=z_{3}-\beta_{2}\varepsilon_{1}+\psi u\\ \begin{array}{c} \dot{z_{3}}=-\beta_{3}\varepsilon_{1}\;\\ \varepsilon_{1}=z_{1}-x_{1}\; \end{array}\; \end{array}\right.$$ (9)

In the equation, $\varepsilon_{1}$ is the error signal of the state observer, $z_{1}$ is the speed observation signal, $z_{2}$ is the observed speed differential signal, and $z_{3}$ is the estimation of the disturbance.

In the application of isokinetic equipment, a discrete second-order linear state extended observer can be obtained (Equation 10):

$$\left\{\begin{array}{c} \varepsilon_{1}=z_{1}\left(k\right)-y\left(k\right)\;\\ \begin{array}{c} z_{1}\left(k+h\right)=z_{1}\left(k\right)+h\left(z_{2}\left(k\right)-\beta_{1}\varepsilon_{1}\right)\;\\ z_{2}\left(k+h\right)=z_{2}\left(k\right)+h\left(z_{3}\left(k\right)-\beta_{2}\varepsilon_{1}+\psi u\left(k\right)\right)\\ z_{3}\left(k+h\right)=z_{3}\left(k\right)-h\beta_{3}\varepsilon_{1}\; \end{array} \end{array}\right.$$ (10)

$\beta_{1}$ , $\beta_{2}$ , and $\beta_{3}$ is the gain of SLESO and is the parameter to be tuned. Based on the bandwidth ω of SLESO, adjusting parameters $\beta_{1}$ , $\beta_{2}$ , and $\beta_{3}$ can reduce computational costs. According to the stability requirements of the observer, the ideal formula of parameter adjustment is: $\beta_{1}=3\omega$ , $\beta_{2}=\frac{3\omega^{2}}{5}$ , and $\beta_{3}=\frac{\omega^{3}}{10}$ (Hao et al., 2021). In practical applications, to maintain a certain stability margin, it is necessary to take $\beta_{3}=\frac{\omega^{3}}{15}$ .

#### 3.2.3 Nonlinear error feedback control

NLEFC adaptively modifies the control gain according to the magnitude of the feedback error. When the error is significant, it decreases the control gain to avoid overshoot and instability. Conversely, when the error is small, it increases the control gain to achieve a swift response. NLEFC combines the velocity transition signals $s_{1}$ and $s_{2}$ provided by TD with the velocity signals $z_{1}$ and $z_{2}$ estimated by SLESO in a nonlinear form to generate an error feedback control variable $u_{0}$ .

Set the nonlinear parameter adjustment function to (Equation 11):

$$F_{fal}\left(e,\alpha ,\delta \right)=\left\{\begin{array}{c} {\left|e\right|}^{\alpha }sgn\left(\delta \right),\left|e\right|>\delta \\ \;\frac{e}{\delta^{1-\alpha }},\left|e\right|\leq \delta \; \end{array}\right.$$ (11)

In the formula, e is the input signal, α is the nonlinear control factor, and δ is the filtering factor.

The nonlinear feedback combination form of NLEFC is as follows (Equation 12):

$$\left\{\begin{array}{c} e_{1}=s_{1}\left(t\right)-z_{1}\left(t\right)\;\\ e_{2}=s_{2}\left(t\right)-z_{2}\left(t\right)\;\\ u_{0}=k_{1}F_{fal}\left(e_{1},\alpha_{1},\delta \right)+k_{2}F_{fal}\left(e_{2},\alpha_{2},\delta \right) \end{array}\right.$$ (12)

In the formula, $e_{1}$ is the speed error signal, $e_{2}$ is the differential signal of velocity error, $u_{0}$ is the output of nonlinear error feedback control, $k_{1}$ and $k_{2}$ are the feedback control gain.

For the problem of overestimation caused by encountering large abrupt signals during the disturbance estimation process, the disturbance estimation value $z_{3}$ is treated with amplitude limiting filtering to avoid overcompensation (Equation 13).

$$z_{3}=\lim\left(z_{3}\right)$$ (13)

The $\lim\left(\right)$ is a saturation function, and its maximum and minimum values are set according to the load-bearing capacity of isokinetic equipment.

Afterwards, feedforward compensation is applied to the observed disturbances to generate the control law of the speed controller (Equation 14):

$$u\left(t\right)=\frac{u_{0}-\lim\;\left(z_{3}\left(t\right)\right)}{\psi }$$ (14)

In the formula, the molecular part is the feedforward compensation result that compensates for model errors and disturbance effects, and the size of ψ reflects the strength of the controller’s compensation for disturbances.

The parameters of NLEFC are set to $\alpha_{1}=0.25$ , $\alpha_{2}=0.75$ , δ=0.05 , $k_{1}=5.6$ , $k_{2}=1$ , and ψ=9 .

#### 3.2.4 Stability analysis

To comprehensively motivate the design of the speed controller, stability proofs for the SLESO and isokinetic control system were completed based on Lyapunov stability theory and Nyquist stability criterion (Castillo et al., 2019; Guo et al., 2018).

Equation 8 and Equation 9, the error dynamic equation of the observer (Equation 15) can be written as:

$$\left\{\begin{array}{c} \varepsilon_{i}=z_{i}-x_{i},i=1,2,3\\ \dot{\varepsilon \;}=A\varepsilon +B\left(\dot{z_{3}}-\dot{x_{3}}\right)\; \end{array}\right.$$ (15)

The observation error matrix $\varepsilon ={\left[\begin{array}{ccc} \varepsilon_{1} & \varepsilon_{2} & \varepsilon_{3} \end{array}\right]}^{T}$ .

$$A=\left[\begin{array}{ccc} -\beta_{1} & 1 & 0\\ -\beta_{2} & 0 & 1\\ -\beta_{3} & 0 & 0 \end{array}\right]$$

***B** =* ${\left[\begin{array}{ccc} 0 & 0 & 1 \end{array}\right]}^{T}$

Configure the poles of SLESO at the bandwidth ω . Therefore, matrix A satisfies the Hurwitz stability condition, and there exists a positive definite Hurwitz characteristic matrix P that satisfies (Equation 16):

$$A^{T}P+PA=-I$$ (16)

Where I is the identity matrix, it can calculate (Equations 17, 18):

$$P=\left[\begin{array}{ccc} -p_{11} & -0.5 & {\;p}_{13}\\ -0.5 & -p_{13} & -0.5\\ \;p_{13} & -0.5 & -p_{33} \end{array}\right]$$ (17)

$$\left\{\begin{array}{c} p_{11}=\frac{\beta_{2}^{2}+\beta_{2}+\beta_{3}^{2}+\beta_{1}\beta_{3}}{2\left(\beta_{3}-\beta_{1}\beta_{2}\right)}\;\\ p_{13}=\frac{\beta_{1}^{2}+\beta_{2}+\beta_{1}\beta_{3}+1}{2\left(\beta_{3}-\beta_{1}\beta_{2}\right)}\;\\ p_{33}=\frac{\beta_{1}^{3}+\beta_{3}\beta_{1}^{2}+\beta_{1}\beta_{2}^{2}+\beta_{3}^{2}+\beta_{1}+\beta_{3}-\beta_{2}\beta_{3}}{2\beta_{3}\left(\beta_{3}-\beta_{1}\beta_{2}\right)} \end{array}\right.$$ (18)

Defining $E=\frac{\varepsilon }{\omega }$ and Lyapunov function $V\left(E\right)=E^{T}PE$ , there is a new error matrix $E={\left[\begin{array}{ccc} E_{1} & E_{2} & E_{3} \end{array}\right]}^{T}$ , which can be calculated as (Equation 19):

$$\dot{V}\left(E\right)=-\omega \left(E_{1}^{2}+E_{2}^{2}+E_{3}^{2}\right)+\frac{\left(2p_{11}E_{1}-E_{2}-2p_{33}E_{3}\right)\left(\;\dot{z_{3}}-\dot{x_{3}}\right)}{\omega }$$ (19)

Since $x_{3}$ is a human-influenced continuous-time varying function, it is bounded within the domain $\left[0,T\right]$ , where T is the running time of the equipment, which is a finite positive real number.

Assuming the desired trajectory of $x_{3}$ is smooth enough such that it is at least second-order differentiable. According to Lagrange mean value theorem, there is the following Equation 20:

$$\left|\dot{x_{3}}\left(T\right)-\dot{x_{3}}\left(0\right)\right|=\left|\ddot{x_{3}}\left(\xi \right)\right|\left|T-0\right|,0<\xi <T$$ (20)

According to boundedness theory, there exists a Lipschitz constant L that holds $\ddot{x_{3}}\left(\xi \right)\leq L$ . In summary, $x_{3}$ satisfies Lipschitz gradient continuity, therefore the following equation holds (Equation 21):

$$\left(\;\dot{z_{3}}-\dot{x_{3}}\right)\leq L\left\|z-x\right\|$$ (21)

Determining the Lipschitz constant L over an arbitrary uncertain time interval $\left[0,T\right]$ when dealing with manually applied random disturbances is challenging. Therefore, we simplified the determination of the Lipschitz constant by utilizing disturbance estimate data obtained from the NLEFC. Specifically, a global Lipschitz constant L is calculated from the dataset in place of the one over $\left[0,T\right]$ .

Equation 19 and Equation 21 with the relationship $2p_{11}E_{1}-E_{2}-2p_{33}E_{3}={2E}^{T}PB$ , it can obtain (Equation 22):

$$\frac{{2E}^{T}PB\left(\;\dot{z_{3}}-\dot{x_{3}}\right)}{\omega }\leq \frac{2LE^{T}PB\left\|z-x\right\|}{\omega }$$ (22)

When ω≥1 , there are $\frac{\left\|z-x\right\|}{\omega }=\frac{\left\|\varepsilon \right\|}{\omega }\leq \left\|E\right\|$ , and the latter equation holds ${\left\|LPB\right\|}^{2}-2\left\|LPB\right\|+1\geq 0$ , then (19) has the following relationship (Equation 23):

$$\dot{V}\left(E\right)\leq -\omega \left(E_{1}^{2}+E_{2}^{2}+E_{3}^{2}\right)+\left({\left\|LPB\right\|}^{2}+1\right){\left\|E\right\|}^{2}$$ (23)

From the above equation, it can be concluded that when $\omega >{\left\|LPB\right\|}^{2}+1$ , $\dot{V}\left(E\right)<0$ , and since ***P*** is a positive definite matrix, it can be inferred that $V\left(E\right)>0$ . According to Lyapunov stability theory, the observer is stable.

The open-loop transfer function of the speed controller can be expressed as (Equation 24):

$$F_{o}=G_{TD}G_{NLEFC}G_{Current\_loop}H_{SLESO}$$ (24)

**FIGURE 5:**
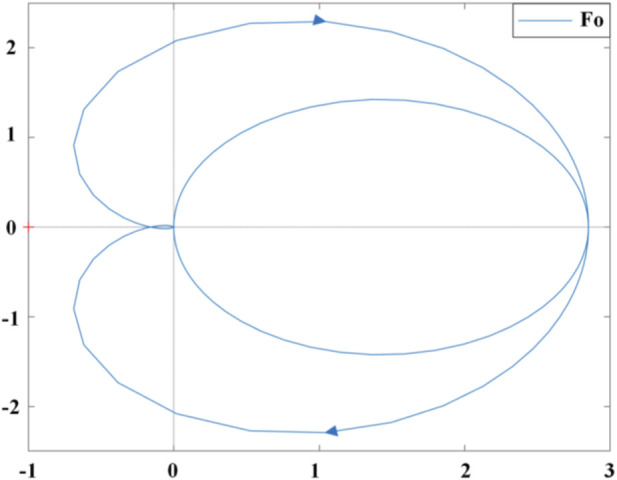
Nyquist curve of the isokinetic equipment control system.

In the formula, $G_{TD}$ is the transfer function of the TD module, $G_{NLEFC}$ is the transfer function of the NLEFC module, $G_{Current\_loop}$ is the transfer function of the current loop controller module, and $H_{SLESO}$ is the transfer function of the SLESO module. From the Nyquist curve of $F_{o}$ shown in Figure 5, it can be concluded that the system is stable.

## 4 Dynamic estimate of torque at the joint level

In the process of isokinetic training, the rotational motion of human joints is usually accompanied by various ancillary movements. The torque measured by the torque sensor not only is the torque applied by the trained joint, but also includes the weight moment of the support rod and arm, the inertia moment affected by angular acceleration, etc. Therefore, it cannot be directly used calculation for torque at the joint level. In order to eliminate the influence of various interference torques other than joint torque and obtain accurate parameters of torque at the joint level, this paper takes the elbow joint as an example, and proposes a dynamic estimate method of torque at the joint level by establishing a human-machine mechanical closed chain model between the isokinetic muscle strength training equipment and the user.

**FIGURE 6:**
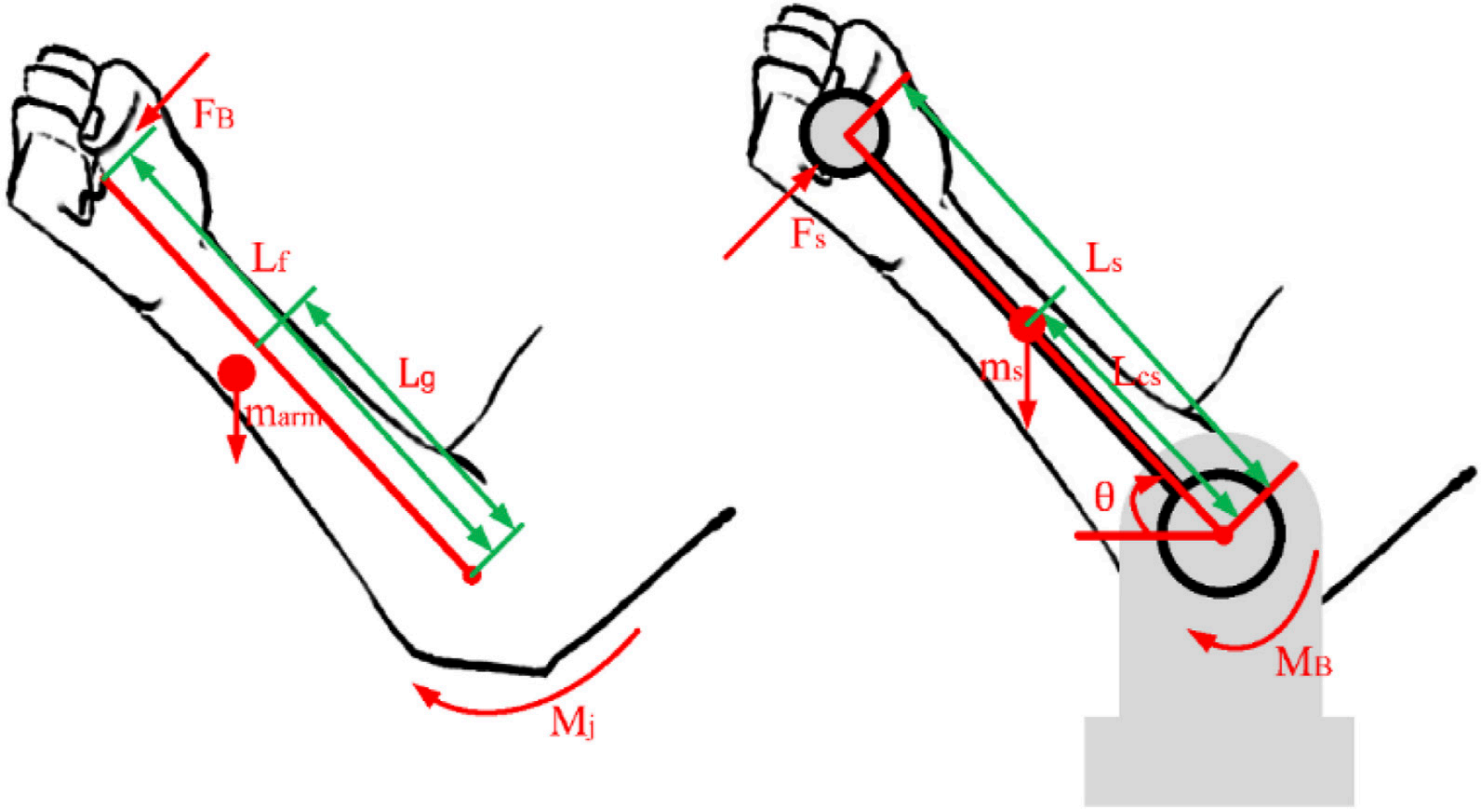
Modeling and analysis of torque at the joint level.

The torque of elbow joint can be decomposed into two rigid body segments, the support rod and the arm, for modeling and analysis, as shown in Figure 6. In calculations, the motion of other planes is ignored since the measured values of gravity and inertia are both in the sagittal plane.

The moment of gravity acting on the supporting rod $M_{Gs}$ is (Equation 25):

$$M_{Gs}=m_{s}gL_{cs}\cos\left(\theta \right)$$ (25)

In the formula, $m_{s}$ is the mass of the supporting rod. $L_{cs}$ is the distance from the center of mass to the center of rotation. θ is the angle between the supporting rod and the horizontal plane.

The moment $M_{Fs}$ of the arm to the supporting rod is (Equation 26):

$$M_{Fs}=F_{s}L_{s}$$ (26)

In the formula, $F_{s}$ is the applied hand thrust. $L_{s}$ is the distance from the grip to the center of rotation.

According to Newton’s second law, there is the following formula (Equation 27):

$$M_{B}-m_{s}gL_{cs}\cos\left(\theta \right)+F_{s}L_{s}=I_{B}\ddot{\theta }$$ (27)

In the formula, $M_{B}$ is the driving torque, $I_{B}$ is the moment of inertia of supporting rod.

A mechanical analysis of the arm can lead to the following formula (Equation 28):

$$M_{j}-m_{arm}gL_{g}\cos\left(\theta \right)-F_{B}L_{f}=I_{j}\ddot{\theta }$$ (28)

In the formula, $m_{arm}$ is the mass of arm. $L_{g}$ is the distance from the arm to the center of mass. $F_{B}$ is the reaction force exerted by the grip on the hand, and $F_{B}=-F_{s}$ . $L_{f}$ is the distance from the center of elbow joint rotation to hand contact grip, and it is approximately equal to $L_{s}$ . $I_{J}$ is the moment of inertia of the arm.

According to Chinese national standard GB/T 17,245-2004, there is a relationship between the mass of arm, height and weight as follows (Equation 29):

$$m_{arm}=-0.701+0.019m_{user}+0.0005H_{user}$$ (29)

In the formula, $m_{user}$ is the weight of user, and $H_{user}$ is the height of user, recorded based on the user’s actual weight and height data.

Calculate the reactive force $F_{B}$ exerted by the supporting rod on the hand using (27) and the principle of reaction force. Substituting it into (28), the torque at the elbow joint level $M_{j}$ is obtained as (Equation 30):

$$M_{j}=I_{j}\ddot{\theta }+m_{arm}gL_{g}\cos\left(\theta \right)+I_{B}\ddot{\theta }-M_{B}+m_{s}gL_{cs}\cos\left(\theta \right)$$ (30)

$M_{j}$ is suitable for speed control algorithms and dynamic estimate of torque at the joint level.

During the training process, the three accessories of this equipment cause the corresponding joints of the human body to rotate about the normal to the sagittal plane. The human-machine mechanical closed chain model established for the shoulder and wrist joints is similar to the elbow joint, therefore the dynamic estimate method of torque at the joint level proposed in this section is applicable to rehabilitation training for other joints.

## 5 Experimental results

To verify the effectiveness of the lightweight upper limb isokinetic rehabilitation training equipment proposed in this paper, this section is based on two key technologies of isokinetic equipment, and tests the feasibility of speed planning strategies and the credibility of dynamic estimate of torque at the joint level through speed control experiments and joint torque estimate experiments.

### 5.1 Speed control experiment

**FIGURE 7:**
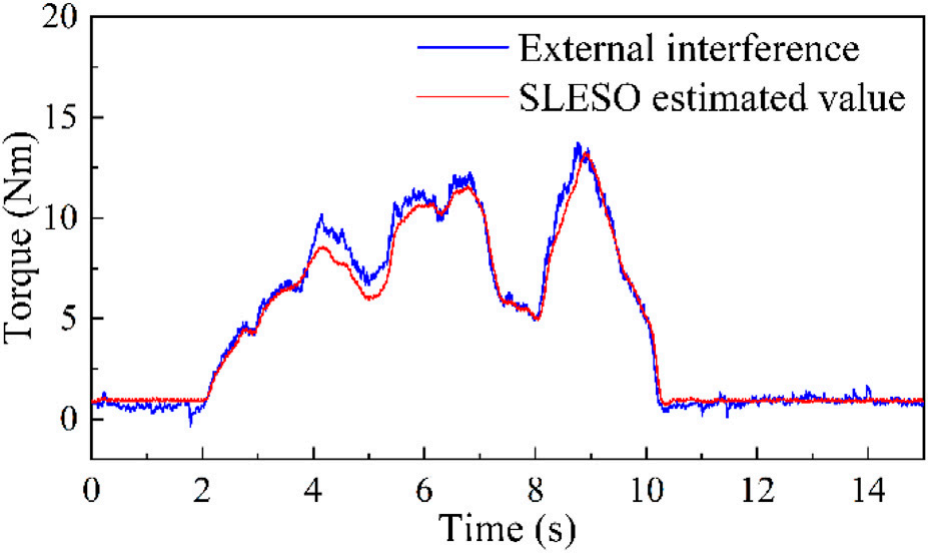
Experiment on SLESO’s ability to estimate disturbances.

Firstly, to ensure the effectiveness of the proposed isokinetic equipment, it is necessary to verify the observation capability of SLESO. In the experiment, the isokinetic equipment is first accelerated to the preset maximum speed for operation, and then manually introduce random time-varying disturbances. At this point, the external disturbance applied is recorded using a torque sensor, while observing the estimation results of SLESO. The experimental results are shown in Figure 7, where the root mean square error (RMSE) between the external disturbance curve and the SLESO observation curve is 0.52. The experiment indicates that SLESO can accurately estimate disturbances.

**FIGURE 8:**
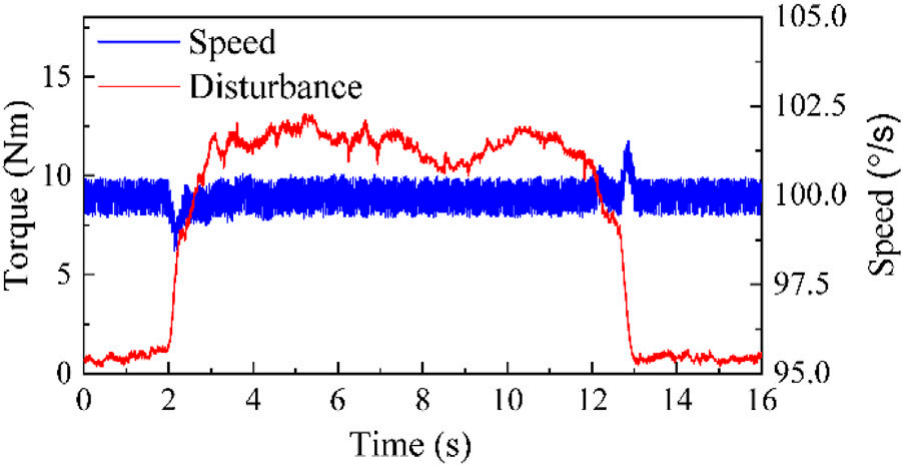
The speed control effect of NLADRC.

**FIGURE 9:**
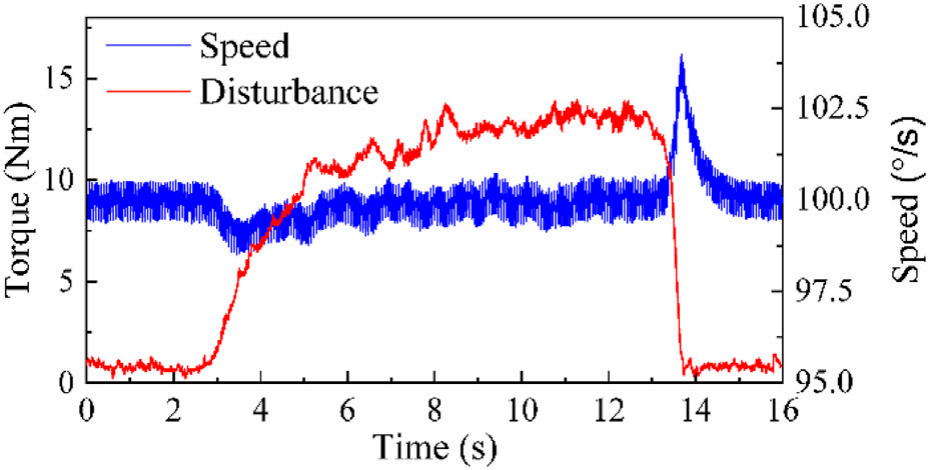
The speed control effect of PID.

In order to further verify the control performance of the proposed algorithm, external torque interference during isokinetic training was simulated and applied to isokinetic equipment, and a comparative experiment was conducted between the NLADRC and conventional PID algorithms. To ensure the fairness of the comparative experiments, based on the principle of controlling variables, the TD, NLEFC, and SLESO modules used in the algorithm of this paper were removed from the original isokinetic control system framework. Instead, a PID control module is incorporated. The PID module utilizes a cascade PID control structure for speed and current to enhance control effectiveness. In this structure, the outer loop employs a PI controller with control parameters $K_{p}=0.0856$ and $K_{i}=0.019$ , and the inner loop adopts a PID controller with control parameters $K_{p}=38$ , $K_{i}=84$ and $K_{d}=5$ . Figures 8, 9 respectively display the speed control effects of NLADRC and PID under external force disturbances.

The experiment displays that the maximum speed tracking errors of NLADRC control algorithm and PID control algorithm are 1.27% and 3.91%, and the maximum asymptotic stability time is 0.423s and 1.255 s. Through comparison, it can be seen that the NLADRC control algorithm has better anti-interference ability and robustness than the PID control algorithm in speed control algorithms.

**FIGURE 10:**
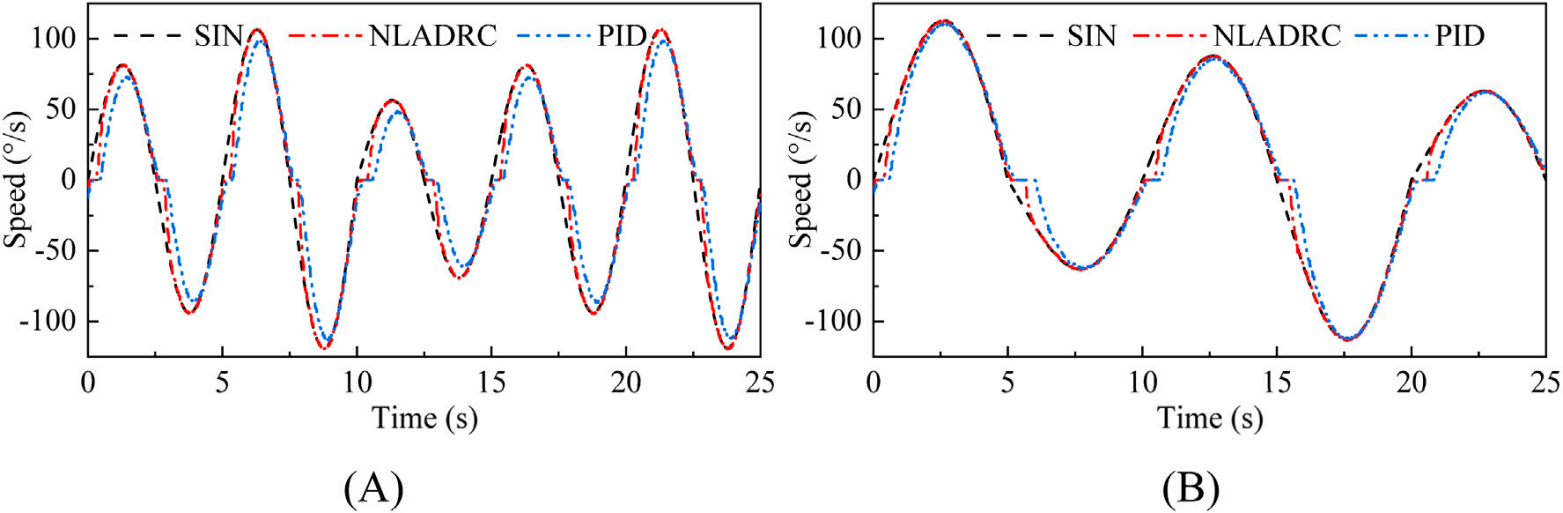
Experimental results of fast and slow sinusoidal speed tracking experiments. **(A)** Fast sinusoidal speed tracking **(B)** Slow sinusoidal speed tracking.

In order to evaluate the speed tracking performance, NLADRC and PID control algorithm were used to conduct fast and slow sinusoidal speed curve tracking experiments. The results are shown in Figure 10, and it can be found that both PID and NLADRC algorithms can achieve convergence of speed tracking.

**FIGURE 11:**
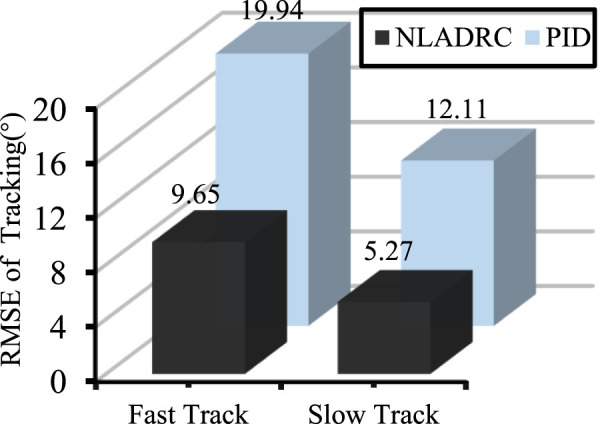
RMSE analysis of tracking at different speeds.

RMSE analysis was conducted on the speed tracking performance of NLADRC and PID algorithms to detect control performance. Figure 11 displays the experimental result. Regardless of whether it is fast or slow motion, the RMSE of NLADRC tracking speed was reduced by more than half compared to PID. Therefore, the designed speed control algorithm has a more ideal speed control effect on isokinetic equipment compared to the PID algorithm.

### 5.2 Isokinetic joint torque estimate experiment

**FIGURE 12:**
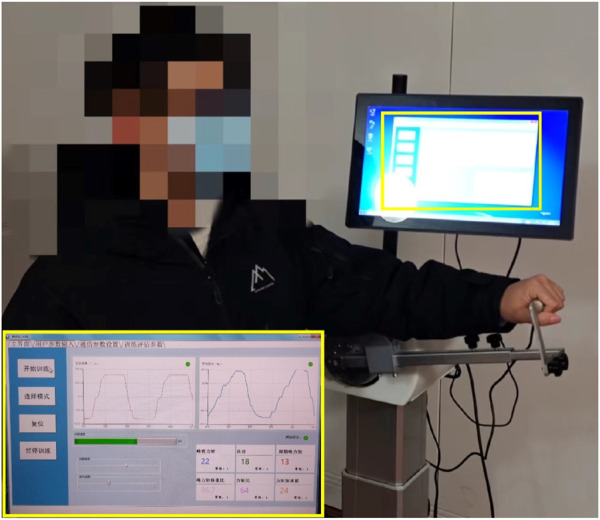
Active isokinetic training experiment snapshot.

In order to experimentally verify the dynamic estimate of torque at the joint level proposed in this paper, ten healthy adults aged between 20 and 44 were selected to participate in the experiment, and all participants expressed their understand and consent. Fix the elbow joint of the test subjects to the isokinetic equipment for active isokinetic training. During the experiment, the subjects are instructed to use their maximum muscle strength as much as possible. The training includes 10 repetitions of flexion and extension movements of the joint at 60°/s, repeated four times. The snapshot in the experiment is shown in Figure 12. Approval of all ethical and experimental procedures and protocols was granted by Shanghai University of Medicine & Health Sciences Ethics Committee under Application No. 2022-ZYXM1-04--420300197109053525, and performed in line with the Declaration of Helsinki.

**FIGURE 13:**
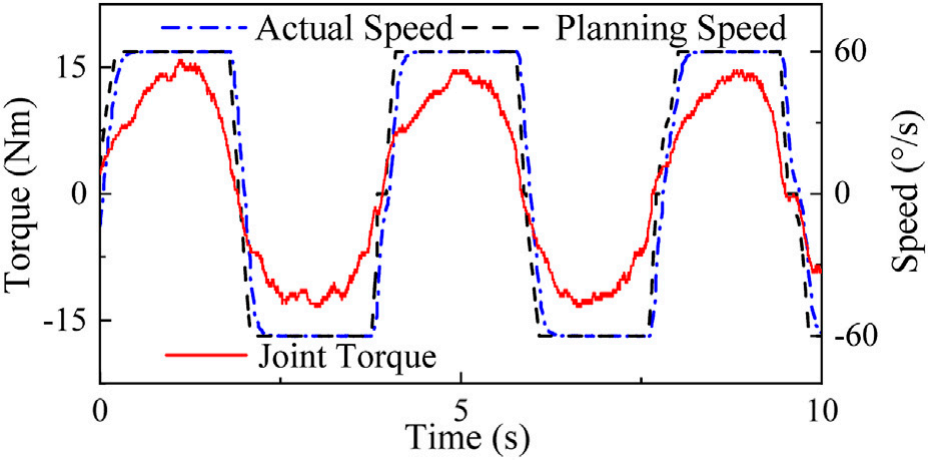
Active isokinetic training experiment.

Figure 13 shows the experimental result. By observing the actual speed curve and joint torque curve, it is evident that the equipment can accurately interpret the user’s movement intention and generate compliant movements within a short time during the acceleration and deceleration stages. Additionally, the joint torque curve shows no significant abrupt changes throughout the entire training process, mitigating the risk of injury. Furthermore, analyzing the speed curve of the complete training cycle reveals that the equipment’s speed approximates the ideal isokinetic motion curve, providing evidence for the effectiveness of the dynamic speed planning strategy proposed in this paper.

**FIGURE 14:**
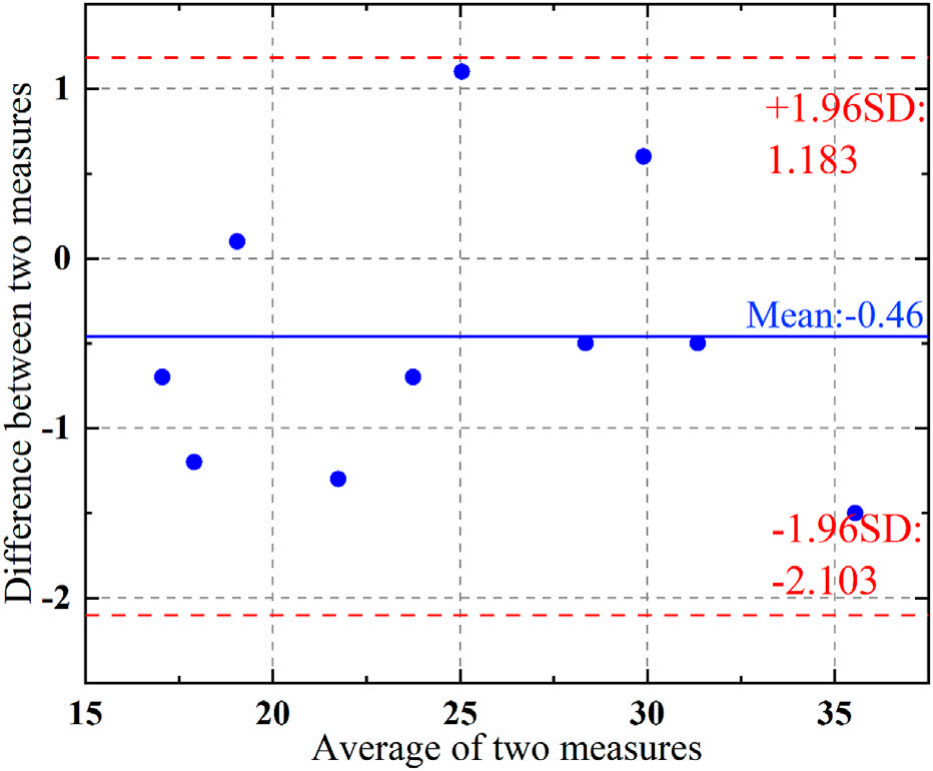
Bland-altman diagram of measurement results.

In order to evaluate the reliability of the dynamic estimate result of torque at the joint level, four test subjects were subjected to active isokinetic training using the Biodex S4 equipment following the same training protocol as described above. Taking the elbow joint as an example, the results obtained from the estimate method used in this paper were compared with the estimate results from the Biodex S4. The body height dimensions of the test subjects and the peak torque test results during the training process are shown in Table 1, and the Bland-Altman consistency test results between the two equipment are shown in Figure 14. According to the consistency test results, the average difference between the measurements of the two equipment is 0.46, with a 95% confidence interval for the difference ranging from −2.103 to 1.183. All measurement data fall within the 95% confidence interval, indicating that the proposed estimate method of torque at the joint level in this paper is effective and has high reliability.

**TABLE 1:** Body height, weight, and peak torque test results for test subjects.

Number	Height (mm)	Weight (kg)	Gender	Method of this paper	Biodex S4
User 1	191	90	Male	34.8	36.3
User 2	172	54	Male	25.6	24.5
User 3	170	59	Male	21.1	22.4
User 4	161	49	Female	16.7	17.4
User 5	182	79	Male	31.1	31.6
User 6	178	75	Male	28.1	28.6
User 7	167	59	Male	23.4	24.1
User 8	174	90	Male	30.2	29.6
User 9	161	55	Female	17.3	18.5
User 10	168	62	Female	19.1	19

## 6 Conclusion

Firstly, this paper has completed the structure design and control system software architecture design of lightweight isokinetic equipment were completed. Secondly, this paper proposes a dynamic speed planning method based on joint torque recognition for motion intention, which improves the safety of rehabilitation training, realizes isokinetic movement of the equipment in response to human movements, and solves the impact problem during the acceleration and deceleration stages of rehabilitation equipment. Once again, this paper investigates the second-order NLADRC speed control method for isokinetic equipment. This method considers the joint torque applied by the user to the isokinetic equipment and the uncertainty fluctuations inside the equipment as disturbances. SLESO is used to estimate the disturbance in real time, and amplitude limiting filtering is used to avoid overcompensation. Subsequently, feedforward compensation is performed to avoid the disturbance from affecting the isokinetic equipment, improved the anti-interference ability and robustness of the speed controller. Next, taking the elbow joint as an example, this paper proposes a dynamic estimate method of torque at the joint level by establishing a human-machine mechanical closed chain model between the isokinetic equipment and the user. Finally, the lightweight isokinetic equipment designed in this paper was tested for overall operation, verifying the feasibility of the software and hardware solutions and the effectiveness of dynamic estimate method of torque at the joint level.

## Data Availability

The original contributions presented in the study are included in the article/supplementary material, further inquiries can be directed to the corresponding author.
